# A cluster randomised feasibility trial assessing an interactive film intervention to improve wellbeing of young people in school settings in the North of England

**DOI:** 10.1186/s40814-025-01699-1

**Published:** 2025-09-02

**Authors:** F. Christie-de Jong, E. Walker, H. Corlett, C. Hardacre, E. Soulsby, B. Arnott, K. Franks, J. Ling, J. Stephenson, L. B. Azevedo

**Affiliations:** 1 School of Medicine, Faculty of Health Sciences and Wellbeing, University of Sunderland, Sunderland, UK; 2 Northeast and North Cumbria Child Health and Wellbeing Network, Newcastle Upon Tyne, UK; 3 Faculty of Health and Education, Manchester Metropolitan University, Manchester, UK; 4 Population Health Sciences Institute, Newcastle University, Newcastle, UK; 5 Youth Focus North East, Gateshead, UK; 6 Faculty of Health Sciences and Wellbeing, University of Sunderland, Sunderland, UK; 7 Department of Allied Health Professions, Sport and Exercise, University of Huddersfield, Huddersfield, UK; 8 School of Sport and Physical Activity, Sheffield Hallam University, Sheffield, UK

**Keywords:** Interactive film intervention, Feasibility study, Young people, Youth engagement, Mental health

## Abstract

**Background:**

Adolescence is a critical period for the onset of mental illness. A partnership of a health and care network and filmmakers developed an interactive film for youth wellbeing. While such films have potential as a cost-effective preventative tool, their effectiveness remains unproven. This study aimed to assess the feasibility and acceptability of a randomised controlled trial of the interactive film intervention to improve wellbeing in school-aged youth.

**Methods:**

In a mixed-methods cluster randomised feasibility trial in North East England (2021–2022), students in years 10 (14–15 years) and 12 (16–17 years) from three schools were recruited and randomised to the following conditions: (1) watching the film in class, (2) watching the film in class with support from youth workers or (3) regular class activities. Feasibility outcomes included willingness of schools to participate, participant recruitment, and retention, which were assessed quantitatively and qualitatively. Data were analysed descriptively and with the use of thematic analysis.

**Results:**

School recruitment targets were met, although this was challenging due to resource constraints and the COVID-19 pandemic. Questionnaires were completed before watching the film by 172 students (48% of the recruitment target). Follow-up targets for retention were met at 3 months (*n* = 138) and 6 months (*n* = 136). Retention of year 10 students was high (96%), but year 12 students had lower retention (60%). Qualitative findings showed students and teachers supported the intervention and trial and measurements; however, consent-taking required more time. Communication and resource issues within schools were challenging and need addressing before moving to a larger trial.

**Conclusion:**

Although some trial aspects were feasible and acceptable, particularly the intervention, others, such as recruitment, retention and school communication, posed challenges. We recommend future feasibility studies should address barriers such as randomisation, communication with schools, recruitment of older students (16–18 years), consent and measurement alignment before moving to a larger-scale trial.

**Trial registration:**

ClinicalTrials.gov Identifier: NCT06807931. Retrospectively registered 04 February 2025 https://clinicaltrials.gov/study/NCT06807931.

## Key messages

- What uncertainties existed regarding the feasibility?
  The effectiveness of this newly developed intervention has not been tested. It is unclear whether testing its effectiveness would be feasible and acceptable, particularly within school settings, due to potential challenges in recruitment, retention and data collection.
- What are the key feasibility findings?
  Progression criteria evaluating key trial parameters—such as recruitment of schools and participants, participant retention, and the acceptability of data collection measures—indicated that progressing to a full trial would be feasible. However, qualitative data and researcher insights into the school context highlighted some challenges that would need to be addressed before proceeding to a full trial. These included logistical barriers and communication issues within the school environment.
- What are the implications of the feasibility findings for the design of the main study?
  Key challenges, such as communication with schools, would need to be addressed before progressing to a full trial. Closer engagement with schools from the outset, ideally through co-production and the appointment of a key contact person within each school, would help resolve some of the challenges encountered. A deeper understanding of the school context will be essential for ensuring smoother implementation and higher engagement in the main study.

## Background

Adolescence is a period of heightened vulnerability for the onset of mental illness, with 75% of all mental health problems established before 18 years old [1, 2]. Mental illness can impact young people’s ability to navigate the stresses of adolescence, leading to isolation, diminished self-esteem, and academic struggles [3]. Unaddressed, these issues may persist into adulthood, becoming more severe and chronic, with consequences for physical health, social adjustment, and economic productivity across the life course [4]. Beyond individual outcomes, early mental health difficulties can have long-term consequences that shape life trajectories, reducing opportunities for education, employment, and social participation, which can ultimately limit the chance to lead fulfilling and independent lives. These cumulative effects contribute to broader social and economic inequalities [4]. In 2020, a UK national survey showed that one in six young people (aged 5–19) had a probable mental health condition—an increase from one in nine young people in 2017 [5]. Rising levels of mental ill health may be partly attributed to increased reporting and awareness. Strategies to tackle what may be framed as a mental health crisis emerge at pace [3, 6, 7] including school-based mental health programmes, counselling services, and de-stigmatisation campaigns. However, adolescents still lack a good understanding of the experience, impact, and management of mental health, which results, as recent systematic reviews show, in negative attitudes towards available support and a reluctance to seek help [8–10]. Film-based interventions may improve mental health literacy because of their potential to engage and be emotionally impactful, which could help young people understand human experiences [8, 11], but the effectiveness of such interventions has been given little scrutiny and there is a lack of evidence on the feasibility of deploying such interventions in schools. Difficulties to implement such interventions include the practicalities of executing interventions in complex, dynamic settings, which can introduce issues with selection, performance, and detection bias. Ethical challenges are also typical in the school environment, particularly when obtaining informed consent or ensuring equitable access to interventions [12]. Finally, health and education research systematically assesses the effectiveness of innovative approaches using randomised controlled trial (RCT) designs [13, 14]. However, this research methodology is troubled by the challenge of conducting work in real-life school environments and needs to be trialled before being implemented on a large scale [15]. Taken together, these challenges require careful consideration and planning to deliver and evaluate the intervention in school settings. Assessing the viability of recruitment, implementation, and measures underscores the significance of feasibility studies as an important preliminary stage for evaluations using RCTs in naturalistic settings [16].

## Aim and objectives

The primary aim of this study was to assess the feasibility and acceptability of a randomised controlled trial of an interactive film intervention aiming to build resilience, enhancing mental wellbeing and help-seeking attitudes for young people (14–18 years) in schools located in deprived areas of the North East and North Cumbria (NENC).

The specific objectives of the study were.

1. To assess the feasibility of delivering a brief interactive film intervention in school settings with a three-arm randomisation at the school level.
2. To explore the suitability of measuring the selected parameters (e.g. recruitment, retention) of the trial with a view to developing a large-scale trial.
3. To explore views and experiences of young people on acceptability and feasibility of the trial and the film intervention through using a qualitative design.
4. To gather preliminary data on the effectiveness of a brief interactive film intervention in school settings to enhance resilience, help-seeking attitudes and mental wellbeing in young people.

## Methods

This study adheres to the CONSORT (Consolidated Standards of Reporting Trials) extension for pilot and feasibility trials [17].

### Ethical considerations

Ethical approval was obtained from the University of Sunderland Research Ethics Group in September 2021 (reference number 009976). Each participating school received a payment of £750 to compensate for the time, effort and resources involved. Students received a £5 retail gift voucher at each quantitative data collection point. Students who participated in focus groups received an additional £5 voucher to acknowledge their time and effort.

### Patient and public involvement (PPI)

To inform the design of this study, we invited young peoples from the NENC region to take part in seven meetings held between August 2021 and September 2022. The number of young people attending each meeting varied between one and eight. Two sessions were originally planned with three participants each, but only one participant was able to attend in both cases. As these sessions were held online and the young person had taken time out to attend, the sessions proceeded, as their input was still considered valuable. These sessions focused on trial design, including recruitment, data collection, and dissemination, as well as feedback on the film. Participants also gained research skills, covering topics including developing research questions and methods.

PPI activities were also conducted towards the end of the study, in July 2023. Creative methods including card sorting and ranking and an effort-impact matrix were used with nine young people to explore key study elements and develop recommendations on developing resources for future research and strategies for sharing findings [18]. The PPI work underwent external evaluation by Investing in Children (IiC), an independent children’s rights organisation based in the North East of England, and achieved the quality standards necessary to receive the Dialogue and Change award. This award acknowledges research projects that actively involve individuals with lived experience, particularly children and young people. This was a pilot scheme that was developed by the funder in collaboration with IiC to evaluate the impact of public involvement and community engagement.

### Study design

A mixed-methods feasibility cluster randomised controlled trial was conducted with three arms to assess the feasibility and acceptability of intervention delivery and study procedures. Progression criteria, including a qualitative process evaluation to provide context [19], were developed to evaluate feasibility and readiness for a definitive trial based on the literature [16], including school recruitment and randomisation (at the *school* level for a cluster-randomised trial), participant recruitment and retention, consent-taking, data collection tools, data analysis, intervention acceptability and delivery.

### Setting

Three schools located in the NENC region and identified by the NENC Child Health and Wellbeing Network, were contacted by telephone and email to engage with head teachers. We aimed to select, from the pool of potential schools, three schools with approximately comparable Mean Socio-economic Status (SES) scores based on the proportion of children entitled to free school meals.

### Participants

Participants were recruited from years 10 (aged 14–15 years) and 12 (aged 16–17 years) from participating schools. Years 11 and 13 were not targeted because of exam preparation, particularly in light of COVID-19 disruption. Schools were asked to identify classes within these year groups. Participant information and consent/assent forms were provided by the schools to students and parents of year 10 students. Year 10 and 12 students who provided written informed consent/assent, and year 10 students who received parental consent, were invited to complete baseline measures. No other eligibility criteria were applied. Since year 12 students were 16 years old and older, they did not require parental consent. For the qualitative evaluation, all participants who had completed the baseline measures were invited to take part in focus groups to share their perspectives on the trial procedures and the intervention’s impact. A researcher explained the purpose of the focus groups during a classroom session, making clear that participation in both the trial and focus groups was voluntary and that individuals could withdraw at any time.

As this was a feasibility study, a formal sample size calculation was not required [20]. Informed by evidence indicating a median sample size of 36 per arm (range 10 to 300) in UK feasibility trials [20], we adopted a pragmatic approach, aiming for a sample size of 120 adolescents per school (i.e. 360 in total). Within each participating school, we aimed for an approximately equal distribution between year 10 and year 12 participants. This target was considered achievable within the study timeline and available budget.

### Study intervention

In 2019, the NENC Health & Wellbeing Network commissioned TryLife to make an interactive film co-produced with young people in 2021. TryLife is an interactive film series that aims to provide young people with a virtual experience of making choices and facing consequences in various scenarios. TryLife is designed to simulate real-life situations and challenges, allowing young people to explore different paths and outcomes based on the decisions they make for the characters in the story. Multiple public health issues are integrated into the films, including mental health and wellbeing. The film series that was commissioned and included in the trial was ‘Jessica’s Story’, which focused on the intersection between young parenthood and perinatal mental health, as well as other public health issues relevant to young people, including domestic violence and help-seeking behaviour.

### Randomisation

The three participating schools were randomly assigned by a researcher using a number generator using block randomisation (with a single block of 3) to one of three intervention conditions:

#### Interactive film (IF)

Participants in this condition engaged in two class sessions wherein they watched the interactive film facilitated by a teacher. The film featured decision points, and the viewer’s choices influenced the storyline’s progression.

#### Interactive film plus youth worker support (IFYWS)

Participants in this condition watched the interactive film as in the previous condition, but with facilitation by a trained youth worker. They engaged in interactive discussions that focused on the decisions made in the film and their potential consequences.

#### Control condition

Participants in this condition received the standard Personal, Social, Health, and Economic (PSHE) Education curriculum provided by the school. PSHE is a school subject in the UK that supports pupils'personal development by teaching them about health, relationships, wellbeing, and financial literacy.

Only one school per arm was included due to the study’s focus on feasibility rather than effectiveness, and was therefore small in scale; a limited budget was also a contributing factor. As such, the findings are not intended to be generalisable but to inform the design of a future, larger trial. Blinding was not considered necessary for a feasibility trial, which focused on assessing the feasibility and acceptability of recruitment, intervention and measurements [17].

### Data collection

#### Primary outcomes

Quantitative and qualitative measurements were used to assess feasibility as primary outcome. These included school recruitment, randomisation, participant recruitment and retention, consent procedures, data collection tools, and perspectives on intervention delivery. Researcher notes were used to record school and participant recruitment and retention. Acceptability for randomisation, consent procedures, measurements and interventions were explored via qualitative interviews with teachers (*n* = 4) and focus groups. Four face-to-face focus groups were conducted with 20 students in total, two in IFYWS, two in IF, but none in the control school. The discussions focused on participants’ perspectives on trial procedures and the intervention’s impact.

Qualitative assessment also included semi-structured interviews conducted via Microsoft Teams or telephone with teachers to assess the acceptability of the trial and the intervention. Three teachers were recruited from the IF school, one from the control, but none from IFYWS. Topic guides aimed to explore the acceptability of the content and delivery of the intervention, as well as the acceptability of the delivery of the research and recommendations for improvement.

#### Secondary outcomes

Preliminary analyses were also performed to summarise the following secondary outcomes including resilience, attitudes toward help-seeking, and mental wellbeing at baseline, 3 months, and 6 months follow-up, assessed via validated questionnaires. Resilience was assessed using the Connor-Davidson Resilience Scale for young adults (10 items [21]). This scale has been developed and validated as a measure of the degree of resilience and for screening participants according to the level of resilience (i.e. high, intermediate or low). Attitudes toward help-seeking were measured via the 10-item Attitudes Toward Seeking Professional Psychiatric Help Scale (ATSPPHS) tool [22]. This tool has been validated and has four sub-scales: recognition of personal need for professional help, tolerance of stigma associated with psychological help, interpersonal openness, and confidence in mental health professionals. Finally, wellbeing was assessed via the 14-item Warwick Edinburgh Mental Wellbeing Scale (SWEMWBS). This scale has been validated and is used in the UK and worldwide [23]. Baseline data were collected in November 2021, 3-month follow-up between March and May 2022, and 6-month follow-up between May and July 2022. Delay in timely follow-up data collection was caused by school holidays, a burst pipe in one school, and difficulty in arranging data collection. The trial ended upon completion of the project's designated funding period.

### Data analysis

Descriptive statistics were employed to summarise the feasibility of recruitment and retention. Secondary outcomes of resilience, attitudes toward help-seeking, and mental wellbeing were summarised using descriptive statistics. Qualitative interviews and focus groups were transcribed verbatim by a professional agency. Data were analysed using thematic analysis by two independent researchers using NVivo software [24].

## Results

Trial feasibility is reported here according to (1) the willingness of schools to participate and be randomised; (2) recruitment, retention and consent; (3) suitability of data collection tools and data analysis; and (4) feasibility and acceptability of the intervention. We also present a summary of questionnaire scores for resilience, help-seeking behaviour and wellbeing for intervention arms and control across the three timepoints. Quantitative and qualitative findings are integrated in a true mixed-methods style [25].

### Willingness of schools to participate and be randomised

**Fig. 1:**
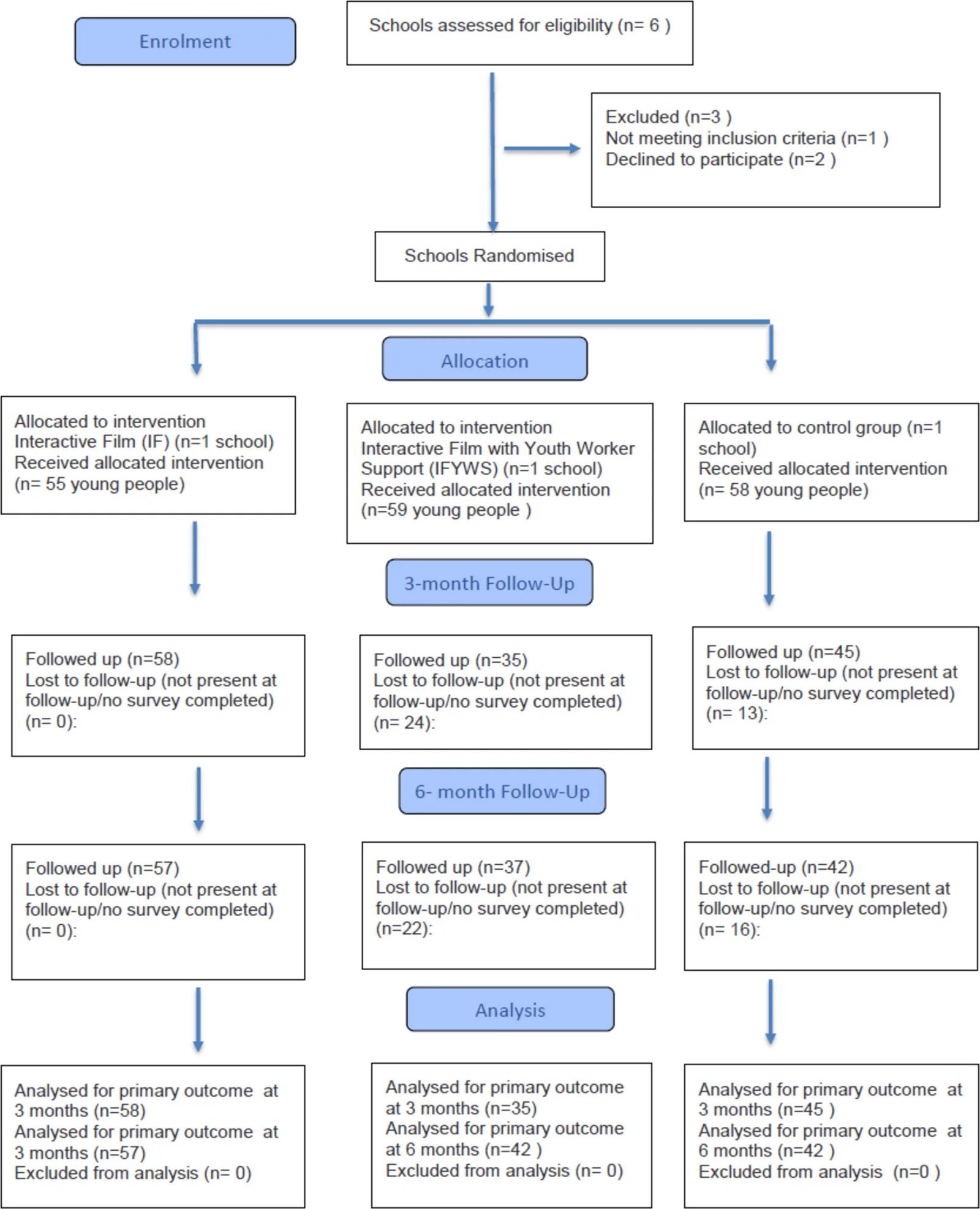
CONSORT flow diagram for cluster (school-based) randomisation

Six schools were directly contacted by telephone or email. Four of these schools were willing to participate and be randomised. Reasons for non-inclusion were non-response after initial contacts (*n* = 2) and teachers’ perception of the school’s inability to facilitate film delivery due to school resources, which meant the school was only willing to be in the control condition and not be randomised (*n* = 1). Hence, three schools were recruited to the project between May 2021 and October 2021. Figure 1 shows flow of eligibility, randomisation and reasons for non-inclusion. Schools from across the North East region and the North Cumbria area (North West region) were targeted for recruitment. However, none of the schools targeted in North Cumbria were able to participate. Hence, participating schools were all in North East England. Schools were diverse in socioeconomic status, with the included schools having 39.2%, 15.4% and 12.2% young people eligible for free school meals, a proxy used for levels of deprivation.

Qualitative data from teachers (*n* = 4) revealed that they believed that project participation was worthwhile, particularly as the study focussed on mental health. Teachers believed the study could be beneficial to students and offered a good opportunity to highlight mental health issues, especially in light of the COVID-19 pandemic and making staff aware of mental health research.

Research itself was also perceived as ‘something very crucial and relevant in education at the moment’ (Teacher 1-IF). Teachers shared they thought participating in research would be a beneficial experience for students and would provide valuable information; for example, for an Extended Project Qualification (EPQ). Teachers also felt that this was an opportunity to contribute to relevant academic research and to ‘*give back’* to the university, which is involved with outreach work with the school.

“Yes, I would take part again. I think as long as the schools see the value, and in this situation, as I said at the start, the value was that the topic, I think, was something very crucial and relevant in education at the moment.”

(Teacher 1-IF)

Qualitative interviews showed that teachers understood that randomisation and control conditions were required in research. Randomisation was seen by teachers as the fairest way to assign conditions. Teachers shared that they would have accepted any condition they were assigned, but noted that watching the video was more exciting for students and would lead to better engagement. Delivery with youth workers would have also been accepted and embraced, as again, it was seen as more exciting for students due to the novelty.

Student participants also had a good understanding of the necessity of a control condition and its role in research and perceived it as the fairest way to assign condition. Although participants in the control condition did not take part in focus groups, participants in the other conditions stated that they would still have participated in the project if assigned to the control condition. However, they shared that being in the control condition might reduce interest and engagement, potentially resulting in attrition. Although the control condition would be acceptable if the group had the opportunity to watch the film afterwards, which was described as a factor aiding willingness to be randomised.

“I think you should do it like that, but then give them the option to watch the film after”

(Student-Y10).

### Recruitment, retention and consent

Two year 10 and two year 12 classes at each school participated, including 172 participants at baseline; 138 at 3-month follow-up; and 136 at 6-month follow-up.

Participant recruitment and retention at baseline, 3-month and 6-month follow-up are summarised by group in Table 1.

**Table 1:** Participant recruitment and retention by group

Timepoint	Variable	Group (study condition)		
		Control	Interactive film	Interactive film with youth worker support
Baseline	Year of study	(*n* = 58)	(*n* = 55)	(*n* = 59)
	Year 10	31 (53.4%)	25 (45.5%)	34 (57.6%)
	Year 12	27 (46.6%)	30 (54.5%)	25 (42.4%)
	Gender			
	Female	52 (89.7%)	32 (58.2%)	40 (67.8%)
	Male	6 (10.3%)	20 (36.4%)	14 (23.7%)
	Other gender identity	0 (0.0%)	2 (3.6%)	3 (5.1%)
	Not recorded	0 (0.0%)	1 (1.8%)	2 (3.4%)
3-month follow-up	Year of study	(*n* = 45)	(*n* = 58)	(*n* = 35)
	Year 10	31 (68.9%)	28 (48.3%)	18 (51.4%)
	Year 12	14 (31.1%)	30 (51.7%)	17 (48.6%)
	Gender			
	Female	44 (97.8%)	35 (60.3%)	25 (71.4%)
	Male	1 (2.2%)	22 (37.9%)	7 (20.0%)
	Other gender identity	0 (0.0%)	1 (1.7%)	1 (2.9%)
	Not recorded	0 (0.0%)	0 (0.0%)	0 (5.7%)
6-month follow-up	Year of study	(*n* = 42)	(*n* = 57)	(*n* = 37)
	Year 10	33 (78.6%)	29 (50.9%)	25 (67.6%)
	Year 12	9 (21.4%)	28 (49.1%)	12 (32.4%)
	Gender			
	Female	38 (90.5%)	29 (50.9%)	26 (70.3%)
	Male	2 (4.8%)	23 (40.4%)	10 (27.0%)
	Other gender identity	0 (0.0%)	2 (3.5%)	0 (0.0%)
	Not recorded	2 (4.8%)	3 (5.3%	1 (2.7%)

Throughout the study, a higher proportion of female than male participants was recruited (72.1% female at baseline), with differences in gender balance across all groups.

Overall, participant numbers dropped by 20% from baseline (*n* = 172) to 3 months follow-up (*n* = 138), but were subsequently practically maintained (a net drop of 1%) at 6 months follow-up (*n* = 136). Attrition varied across groups. The control group experienced moderate attrition between baseline and 3-month follow-up (23% decrease), and minimal attrition between 3-month and 6-month follow-up (7% decrease). The IFYWS group experienced larger attrition between baseline and 3-month follow-up (41% decrease), and a small increase from 3 to 6-month follow-up (5% increase). The difference in attrition rates is out of expectation and suggests disparities in the level of engagement of the included schools over time. Table 1 shows that attrition loss occurred primarily in year 12, with substantial attrition, particularly from 3-month to 6-month follow-up.

Interviews with teachers reported that most students in their class did participate in the project. Teachers suggested that some students might have decided not to participate if they did not fully comprehend the purpose of the study. Teachers thought that students’ interest increased throughout the project as they heard other students discuss the film and teachers felt that there was some disappointment from students who did not take part, that they had missed out when the film was discussed in class.

“There were a few students at the beginning that didn’t consent. They weren’t interested in taking part. I think probably because again they didn’t know what it was about. But I think, as it went through and as they were talking to other people in the group and they saw bits of the video and things, I think the interest level increased after that”

(Teacher 2-IF)

Teachers suggested that more information at the project start may have led to better engagement, and noted that some male students did not want to participate. Teachers suggested that this might be due to ‘teenage ego,’ describing it as some male students declining to participate precisely because most other males were not participating. Teachers also suggested that the film might have been ‘too young’ for year 12 students and may be more suitable for year 10 students, who were 14–15 years of age. In focus groups, students expressed their motivation to participate because they recognised the importance of mental health research. To further enhance participation, they recommended emphasising practical applications of the research.

Qualitative interviews also explored ethical considerations, including consent-taking. Participants stated that they were aware that they had a choice, that participation was voluntary and that they could say no to taking part, which a few did. Some students shared that they were sufficiently informed, although others stated that they would have liked more information, for example what the content of the film would be, and what would be involved:

“Yeah, before we had these sheets, we had no clue about the video or whatever. I didn’t know anything, to be honest”

(Student Y12).

Many of the participants saw the £5 voucher they received as a ‘thank you’ or reimbursement rather than an incentive. Teachers stated the £5 voucher given to students each time they completed a set of questionnaires was really appreciated by the student and made them ‘feel valued’ and showed ‘their time was being rewarded’. The vouchers were seen by teachers as ‘very generous’ and it was suggested that perhaps it was unnecessary to provide a voucher every time.

### Suitability of data collection tools and data analysis

Response rates achieved for all three questionnaires were high, with full participation by over 95% of participants. Response rates for the individual questionnaires were 97.5% for the wellbeing questionnaire, 96.2% for the resilience questionnaire and 95.5% for the help-seeking questionnaire. Participation rates are summarised in Table 2.

**Table 2:** Questionnaire response rate

Measure	Questionnaire completion status		
	Completed	Partially completed	Incomplete
Wellbeing (WEMWBS)	435 (97.5%)	10 (2.2%)	1 (0.2%)
Resilience (CD-RISC)	428 (96.2%)	15 (3.4%)	2 (0.4%)
Help-seeking (ATSPPHS)	426 (95.5%)	11 (2.5%)	9 (2.0%)

^Questionnaires were considered fully complete when every question was answered, ‘partially complete’ when at least 80%of the questions were answered and incomplete where < 80%of questions were answered (2 or more missing responses)^

Qualitative data suggest that questionnaires were acceptable to both teachers and students, with most not minding completing them, and a few even expressing enjoyment in doing so. The data collection procedure was seen as well-organised, easy to facilitate, and acceptable to all. The timing and questionnaire length, as well as the number and clarity of questions, were perceived to be acceptable. A teacher mentioned that students had the same query each time about one particular question in the help-seeking questionnaire. However, questions about the questionnaire were directed to the researcher, who offered support. Teachers shared that organisational procedures made it easy to hand back completed questionnaires as required:

“None of them [questionnaires] [has] taken long, so they’re all right”

(Student Y10)

Follow-up questionnaires were perceived as useful, as participants knew what to expect. Questionnaires were also seen as a platform to talk about mental health. However, some students suggested that classmates might not have completed questionnaires because they found questions too personal. Some participants also questioned the questionnaires’ relevance in relation to the film, with some participants unsure if the questions asked and film topic matched.

“I don’t think they were relevant to the film”

(Student Y12)

### Feasibility and acceptability of the intervention

At baseline, participants were asked if they had heard of TryLife and its interactive films before the study, and if they had watched the specific intervention film, ‘Jessica’s story’ (Table 3). The majority (95.9%) had not heard of TryLife, and nearly all (98.8%) had not watched ‘Jessica’s story’.

**Table 3:** Awareness of TryLife and interactive film

Timepoint	Variable	Group (school type)		
		Control	IF	IFYWS
Baseline	Heard of TryLife			
	Yes	4 (6.9%)	1 (1.9%)	2 (3.4%)
	No	54 (93.1%)	52 (98.1%)	56 (96.6%)
	Watched *Jessica’s Story*			
	Yes	1 (1.7%)	0 (0.0%)	1 (1.7%)
	No	57 (98.3%)	52 (100.0%)	57 (98.3%)

Qualitative data show that there were generally no concerns about the intervention, and it was seen as a straightforward process. Initially, we expected that participants would watch the film individually on a school computer. However, the main obstacle to intervention delivery was the lack of access in schools to the technology required to show the film. In IFYWS, this was overcome by watching the film as a group with one big screen for year 10 and allowing pupils in year 12 to watch the film on their personal phones, individually. In IF, classes were selected based on the room they were in and what technology would be available there. Teachers highlighted challenges with timetabling, suggesting that additional lead-in time could have been beneficial; however, this was not perceived as a barrier to participation.

“So that was the only issue I had, logistically, because our academy students are not meant to use their phones. They’re meant to be on silent, and put away, and they’re not used in the daytime”

(Teacher 3- IF).

The film’s content was also perceived as acceptable by both teachers and students, who enjoyed the film and particularly the discussions that followed. They said that the film fitted the curriculum and helped to raise awareness of issues involved in teenage pregnancy. Some students could see how other topics such as mental health, relationships and domestic abuse were intertwined, although not all could see the relevance of the topics. Most young people in the study sample thought the film was a valuable resource for use at school. However, they could not see themselves interacting with it at home, due to discomfort discussing these topics in the home environment.

### Feasibility summary

Trial feasibility was assessed across four main parameters using project monitoring data collected throughout the project, and according to predefined progression criteria, with a summary provided in Table 4 and the full version in Appendix 1. Progression criteria were assessed against the target of full (green), partial (amber), and non-achievement (red) to provide an in-depth understanding of what worked well and what did not. The full target was met for the feasibility of recruiting schools, participant retention, and acceptability of the intervention and its delivery mode. A partial target was met for school randomisation, participant recruitment, acceptability of consent procedures, secondary outcomes, and data collection methods.

**Table 4:** Summary progression criteria outcomes

Feasibility outcome	Full (green)	Partial (amber)	Non-achievement (red)	Outcome
Recruitment-schools	3	< 3 before baseline	< 3	Green (3 schools recruited)
Randomisation	Yes	Some issues	No	Some issues (1 school not willing to be randomised)
Recruitment-participants	≥ 60%	40–60%	< 40%	Amber (49% recruited)
Follow-up 3 and 6 months	≥ 60%	40–60%	< 40%	Green (80% at 3 months, 79% at 6 months)
Acceptability consent procedures	Yes	Some issues	No	Amber (some issues identified)
Acceptability data collection				
• Response rate	≥ 70%	60–70%	< 50%	Green (95% response rate)
• Qualitative feedback	Yes in all schools	Some issues	No	Amber (some issues)
Acceptability intervention	Yes	Some issues	No	Green

### Preliminary analyses changes in resilience, help-seeking behaviour and wellbeing

Preliminary findings on outcomes presented are indicative only and are not designed to be used to draw conclusions about the efficacy of the intervention due to the small sample size. Resilience, help-seeking behaviour, and wellbeing questionnaire scores are summarised in Table 5. Higher scores indicate greater resilience, better mental wellbeing and more positive attitudes toward professional help seeking.

**Table 5:** Mental wellbeing, resilience and help-seeking attitudes (mean (SD)), at group and timepoint

Timepoint	Variable	Group (school type)		
		Control	IF	IFYWS
Baseline	WEMWBS total score	(*n* = 58)	(*n* = 55)	(*n* = 58)
		42.2 (8.6)	43.5 (10.1)	42.4 (8.7)
	CD-RISC total score	(*n* = 58)	(*n* = 53)	(*n* = 59)
		19.2 (7.1)	20.9 (6.5)	21.3 (7.6)
	ATSPPHS total score	(*n* = 58)	(*n* = 54)	(*n* = 58)
		13.8 (4.8)	14.6 (5.4)	12.8 (5.6)
3-month follow-up	WEMWBS total score	(*n* = 45)	(*n* = 58)	(*n* = 35)
		40.3 (7.8)	44.5 (8.9)	42.3 (9.5)
	CD-RISC total score	(*n* = 45)	(*n* = 58)	(*n* = 35)
		18.6 (6.6)	22.6 (7.2)	22.0 (6.6)
	ATSPPHS total score	(*n* = 44)	(*n* = 56)	(*n* = 35)
		14.2 (4.5)	13.7 (4.8)	12.7 (4.8)
6-month follow-up	WEMWBS total score	(*n* = 42)	(*n* = 57)	(*n* = 37)
		43.5 (8.1)	44.3 (10.0)	42.7 (8.8)
	CD-RISC total score	(*n* = 42)	(*n* = 57)	(*n* = 37)
		21.4 (6.7)	23.9 (7.9)	20.2 (6.3)
	ATSPPHS total score	(*n* = 40)	(*n* = 57)	(*n* = 35)
		13.4 (5.5)	13.6 (6.1)	11.7 (5.8)

## Discussion

The results from this feasibility study indicated that three of the seven progression criteria offered a strong indication to proceed (full target achieved), four showed a medium indication (partial target achieved), and none raised concerns about moving forward (non-achievement target). These findings suggest that it is feasible to recruit schools, retain participants, that the intervention and its implementation were acceptable, and that the use of data collection measurements were feasible. However, before conducting a fully powered randomised controlled trial, findings also suggest that certain methods, such as school randomisation, participant recruitment, clarity of consent procedures, and the applicability of outcome measures, must be reviewed.

While we successfully recruited the required number of schools, this recruitment was challenging, and there was no surplus, leaving no flexibility had any schools withdrawn. Although one participating school had some existing links with the hosting University through outreach activities, these activities had been delivered by a different team and were unrelated to the research. While this prior connection may have provided some familiarity, it is unlikely to have directly influenced the school’s decision to participate. Nonetheless, this context should be considered when interpreting the feasibility of school recruitment in this study.

Lack of resources to deliver the film might have acted as a barrier to randomisation. In this feasibility study, intervention delivery was adjusted to a certain extent to schools’ capability of delivering the intervention (e.g. participants used their phones to watch the film). However, these adjustments were not considered at the initial recruitment stage. In a review, school resources have been highlighted as an issue for delivering mental health promotion interventions in schools [26], suggesting that interventions must adapt to school culture and resources, while supporting the outcome benefits. However, in our study, although school resources affected one school’s willingness to be randomised, this did not impact recruitment as we reached our sample target.

We had aimed to match schools in terms of SES as there is a relation between young people’s mental health and deprivation [27]. However, some diversity between schools’ SES was observed in our study, which used a pragmatic approach. Exploring a gradient of school SES might be needed to understand the acceptability of the intervention, since there is evidence that mental health interventions might need to be adapted to target low-SES populations, including booster sessions [28].

Recruitment of participants was around 50% of our target, with a higher proportion of females in all groups. A few students believed that some of their peers chose not to participate in the study because of a lack of understanding of the study. Teachers also shared that the study’s details could have been clarified verbally in greater depth. This is supported by the qualitative data with students which indicated some study participants wanted more in-depth information about the video and the study. Although every effort had been made to produce easily accessible and age-appropriate documentation to explain the study, crucial to informed consent [29], time for researchers to verbally explain the study was limited. Data collection, including obtaining consent, was constrained by the tight school timetable, leaving researchers dependent on the availability and cooperation of teachers. Logistical challenges during a school-based intervention, including time constraints, have been found elsewhere [30]. Nevertheless, teachers thought that the way that student recruitment was conducted was appropriate, and students who agreed to participate wanted to continue and engage with the study.

Although baseline recruitment did not reach the pre-specified target, retention of participants at 3- and 6 months was adequate, meeting the progression criterion. The IF group surprisingly slightly increased from baseline to 3 months (5% increase), possibly due to participants consenting to participate but not attending baseline data collection. As data were collated and analysed by groups, it could not be known who these individuals were, which is a study limitation. However, in feasibility studies, analysing data at group level rather than individual level can be appropriate [31]. The attrition rates varied according to the study arm, being higher in IFYWS. Reasons for higher attrition in this particular group were not explored but could be related to intervention acceptability or measurements for this specific school due to cultural or environmental factors and impacting the internal validity. However, given that there was only one school in each arm, the most likely reason is chance. In future studies, different attrition rates between intervention arms should be further explored through qualitative methods, and analysed using statistical approaches such as intention-to-treat and multiple imputation [15].

There was a drop in participant numbers in the first 3 months, primarily among year 12 students who were preparing for or discontinued their A-levels. In hindsight, choosing year 12 may therefore not have been suitable. Researchers need to address the logistical challenges of working with schools by implementing robust procedures to gather information on school scheduling and curricula before the study starts [32]. Establishing open communication with a designated staff member who had protected time to support the research project would help address logistical challenges as they emerge [33].

Regarding acceptability of measurements, participants found questionnaires relatively easy to understand and low burden, contributing to a high response rate (over 95%). Teachers viewed the measurements as a potential platform for discussion about broader mental health issues. However, some students questioned the questionnaires’ relevance to the intervention. The questionnaires covered help-seeking, resilience, and wellbeing, while the film (intervention) aimed to raise awareness of young parenthood. While help-seeking, resilience, and wellbeing were underpinning themes in the film, its primary focus on young parenthood may have obscured these underlying issues for some viewers. Given more time and different circumstances, it might have been possible to align the measures more closely with the intervention’s focus. However, pandemic-related delays in the film’s production, communication challenges, and the selection of study outcomes and data collection measures before film completion posed significant constraints. Future interventions should tailor outcomes more specifically to mental health issues targeted by the intervention.

The intervention’s content, particularly the follow-up discussion, was seen as positive by students, and suggestions for addressing other mental health issues could use a similar approach. However, participants indicated they preferred that this type of intervention be delivered at school rather than at home because they felt some discomfort discussing these topics with parents. It has been reported that dropout rates in mental health interventions at home are significantly higher compared to a school intervention [34]. Therefore, school settings might be ideal for intervention delivery of this type. Students enjoyed the intervention delivery by youth workers; however, they also found teacher delivery acceptable. A systematic review found no difference in outcomes between teacher and external intervention delivery, with students appreciating both the relatability of external facilitators and the trusted presence of teachers [15, 35]. Considering scalability, teacher delivery may be a viable option.

Researchers were concerned about the risk of contamination of the control group, since the TryLife films were in the public domain when the study started. However, only one control participant was aware of the intervention, indicating that the dissemination of TryLife should be intensified to reach the target population.

The strengths of this study include use of a mixed-methods approach and validated questionnaires. However, this study has some limitations inherent to feasibility studies, such as limited statistical power and imbalances in baseline scores, influenced by differences between schools, such as the gender imbalance in participating classes. Future research could use targeted strategies to recruit more male students. Our approach to determining the sample size was pragmatic, informed by evidence available at the time of proposal development, which reported a median of 36 participants per arm (range 10–300) in UK feasibility trials [20]. While this offered a useful benchmark, we acknowledge that more recent guidance [36]) offers a more structured, progression-criteria-driven approach to sample size justification in feasibility studies, which could have strengthened our work and may be particularly valuable in informing a future definitive trial.

Particular contextual factors also affected project feasibility. Data collection started in November 2021, when students were just returning to school after the COVID-19 lockdown. Although students or teachers did not mention this during interviews, conducting the study was challenging, largely due to pandemic-related delays and the significant impact on schools still recovering from the pandemic. Communication issues with schools, staff turnover, and unplanned absences, both in schools and within the research team, created additional obstacles. Key contacts in schools left their roles, and illness among school staff as well as the research team further reduced available personnel, straining the team and hindering progress. The difficulties of conducting research within the complex environment schools had to operate in during and after the pandemic are well documented [37]. Recommendations for future studies include improved communication with schools and ensuring a dedicated teacher is allocated sufficient time to support the project. Co-production and ensuring key stakeholders from within all recruited schools are involved from the start, including study design and planning, may have prevented some of the issues.

Other recommendations include providing additional support to schools with the technology required to deliver the project and adopting a flexible approach to deliver the intervention to suit the school’s needs. However, the “active ingredients” of the intervention should be maintained, such as the discussion following the interactive film delivery, and the role of youth workers should be explored further in follow-up studies. Other recommendations include the use of a variety of interactive films to tackle different mental health problems faced by adolescents and involve teachers and students in the selection of mental health issues. The intervention needs to be trialled in more school settings, perhaps investigating more in-depth intervention feasibility in schools at different socio-economic levels. Likewise, measurement tools must match what has been delivered in the intervention, as highlighted by students and reported in the preliminary data analysis.

In conclusion, while the intervention appears feasible and acceptable, its effectiveness as a tool for supporting wellbeing, resilience, and help-seeking behaviour requires further investigation. Conducting a school-based RCT is possible but challenging, particularly in the aftermath of the pandemic, which created barriers not fully captured in the feasibility data. Four out of seven progression criteria were classified as ‘partial target achieved’, indicating a medium likelihood of success in moving to a definitive trial.

Unforeseen issues arose during the feasibility study that could not have been foreseen, and could not be corrected during the conduct of the study. Given the challenges faced—especially with randomisation, communication, and alignment of measurement tools—we recommend pausing before proceeding to a definitive trial. Future feasibility studies should focus on improving communication with schools, involving schools more in the design of studies and aim for co-production, recruitment and consent processes, and developing measurement tools that align closely with the intervention’s targeted behaviours. Addressing these issues will create a stronger foundation for eventual implementation and evaluation.

## Data Availability

Data and materials are available upon reasonable request.

## Appendix 1

**Table 6:** Progression criteria full text

Criterion and method	Full, partial, and non-achievement target	Target met?
Criterion: Was it feasible to recruit schools? Method: Project monitoring data (number of schools and time of recruitment)	Full: Target number of schools (3) recruited before baseline data collection	Full target met: contact was made with seven schools of which three schools were recruited between May and October 2021.
	Partial: Target number of schools (3) not recruited *before *baseline data collection date (but is achieved overall)	
	Non-achievement: full or partial recruitment targets not achieved and recruitment method and eligibility need careful review before progressing to full scale trial.	
Criterion: Were schools willing to be randomised? Method: Qualitative assessment	Full: All schools willing to take part in the study were willing to be randomised.	Partial target met: three schools were willing to be randomised and qualitative analysis of teacher interview and student focus groups suggest there is a good understanding and acceptable of necessity of randomisation for RCT. However, due to lack of resources to show the film, a fourth school that was initially contacted could only take part if assigned to control condition and was therefore not selected to participate.
	Partial: Some schools participating in the study were willing to be randomised, but randomisation was a barrier to participating for others.	
	Non-achievement: full or partial target not achieved, recruitment targets not achieved. Randomisation was a key barrier to non-participation. Recruitment method need careful review before progressing to full scale trial.	
Criterion: Was it feasible to recruit participants? Method: Quantitative descriptive data	Full: In each school and for each arm, at least 60% of participant recruitment is achieved within 6 months [38].	Partial target met: Original target was 120 participants in each school. In each school 48-49% of this target was achieved.
	Partial: In each location and for each arm, between 40% and 60% of participant recruitment is achieved within 6 months.	
	Non-achievement: full or partial target not achieved, recruitment targets not achieved and recruitment method and eligibility need careful review before progressing to full scale trial.	
Criterion: What proportion of participants could be followed up at 3 and 6 months? Method: Quantitative descriptive data	Full: In each school and for each arm, at least 60% of participants could be followed-up at 3 and 6 months [29].	Full target met: participant numbers dropped by 20% from baseline to 3 months follow-up, but were subsequently practically maintained (a net drop of 1%) at 6 months. (80% retention rate at 3 months and 79% retention rate at 6 months).
	Partial: in each location and for each arm, between 40% and 60% of participant could be followed-up at 3 and 6 months.	
	Non-achievement: full and target not achieved, less than 40% of participants could be followed-up at 3 and 6 months, retention and attrition need careful review before progressing to full scale trial.	
Criterion: Were consent procedures acceptable to participants? Method: Qualitative data	Full: In each school, consent procedures were acceptable to all participants, teachers, and parents and no issues raised	Partial target met: qualitative data suggested consent procedures acceptable to participants however, some participants indicated they did not have a complete understanding of the project when signing consent.
	Partial: qualitative data suggested consent procedures were acceptable to most participants, teachers, and parents, although some issues were raised	
	Non-achievement: full or partial target not achieved, qualitative data suggested consent procedures were not acceptable to participants, teachers, and parents.	
Criterion: Were the selected secondary outcomes and data collection methods acceptable to participants and stakeholders? Method: Quantitative descriptive data and Qualitative data	Full: student response rate > 70% at baseline and follow up [29]. In each school, participants and teachers express all data collection process and methods to be acceptable.	Partial target met: response rates achieved for all three surveys implemented in the study were high, with full participation by over 95% of participants. Surveys were seen as straightforward and most questions were perceived as clear, very few were difficult to comprehend. However, some participants questioned the relevance of the surveys.
	Partial: Student response rate > 60% at baseline and follow-up. In at least two schools, participants and teachers express most data collection process and methods to be acceptable.	
	Non-achievement: Student response rate < 50% at baseline and follow up. amber target not achieved. Participants shared major concerns about data collection methods, which need careful review before proceeding to full scale trial.	
Criterion: Was the intervention and its implementation acceptable to participants and stakeholders, including intervention components and delivery mode? Method: Qualitative data	Full: in each school, intervention content and delivery were predominantly acceptable to all participants and teachers.	Full target was met. Qualitative data suggested participants and teachers were positive about the intervention, particularly about the delivery with youth workers and the discussion resulting from this
	Partial: intervention content and delivery were mostly acceptable to participants and teachers, but some issues were raised.	
	Non-achievement: participants and teachers shared major concerns about intervention delivery, content or modality, which need careful review before proceeding to full scale trial.	

Full target achieved: Very strong indication to proceed to a full trial. Partial target achieved: Medium indication to proceed to a full trial. Further discussions need to take place with members of the research team, PPI and stakeholders to improve performance. It should be reviewed in context of relevant qualitative and quantitative data provided in this feasibility study. Non-achievement target: Indication of doubt as to whether to proceed to a full trial. Further discussions need to take place with members of the research team, PPI and stakeholders, review in context of relevant qualitative and quantitative data provided here. Should only proceed if other indicators are full or partial and there is a clear strategy to improve performance of indicators
