# Correction: A cluster randomised feasibility trial assessing an interactive film intervention to improve wellbeing of young people in school settings in the North of England

**DOI:** 10.1186/s40814-026-01964-x

**Published:** 2026-11-11

**Authors:** F. Christie‑de Jong, E. Walker, H. Corlett, C. Hardacre, E. Soulsby, B. Arnott, K. Franks, J. Ling, J. Stephenson, L. B. Azevedo

**Affiliations:** 1 School of Medicine, Faculty of Health Sciences and Wellbeing, University of Sunderland, Sunderland, UK; 2 Northeast and North Cumbria Child Health and Wellbeing Network, Newcastle Upon Tyne, UK; 3 Faculty of Health and Education, Manchester Metropolitan University, Manchester, UK; 4 Population Health Sciences Institute, Newcastle University, Newcastle, UK; 5 Youth Focus North East, Gateshead, UK; 6 Faculty of Health Sciences and Wellbeing, University of Sunderland, Sunderland, UK; 7 Department of Allied Health Professions, Sport and Exercise, University of Huddersfield, Huddersfield, UK; 8 School of Sport and Physical Activity, Sheffield Hallam University, Sheffield, UK

**Correction: Pilot Feasibility Stud 11, 117 (2025)**

**https://doi.org/10.1186/s40814-025-01699-1**

Following publication of the original article, the authors identified an error in the author name of C. Hardacre.

The incorrect author name was: C. Hardarce

The correct author name is: C. Hardacre

The author group has been updated above and the original article [1] has been corrected.
